# Predicting readmission rates in critically ill heart failure patients during a 90-day vulnerable phase using interpretable machine learning models

**DOI:** 10.1016/j.clinsp.2025.100775

**Published:** 2025-09-11

**Authors:** Meng-Han Jiang, Fang Yu, Hai-Ying Yang, Sun-Jun Yin, Li-Juan Yang, Yu Chen, De-Min Li, Yu Guo, Jia-De Zhu, Wen-Ke Cai, Gong-Hao He

**Affiliations:** a Department of Clinical Pharmacy, The 920th Hospital of Joint Logistics Support Force, PLA, China; b College of Pharmacy, Dali University, Dali, China; c Department of Cardiology, The 920th Hospital of Joint Logistics Support Force, PLA, China; d Department of Cardiothoracic Surgery, The 920th Hospital of Joint Logistics Support Force, PLA, China

**Keywords:** Readmission, Heart failure, Deep learning, SHAP, MIMIC

## Abstract

**Objective:**

The readmission rate of critically ill Heart Failure (HF) patients remains high during the vulnerable phase. However, predictive models based on more comprehensive comorbidities and medication histories are lacking. This study aims to extract these factors to develop interpretable models for predicting readmission risk.

**Methods:**

The authors recruited critically ill HF patients from the MIMIC-IV database (as training and internal validation cohorts) as well as from the MIMIC-III database (as external validation cohorts). Four models, including Neural Multitasking Logistic Regression (NMTLR) were constructed and evaluated. Furthermore, Shapley Additive Explanations (SHAP) interpreted feature importance, simplifying the optimal model based on variable importance.

**Results:**

A total of 12,126 patients were included in this study. Among four predictive models, the NMTLR model demonstrated the best predictive performance with the Area Under the Curve (AUC) of 0.752 (internal) and 0.785 (external), with the C-index of 0.7408 (internal) and 0.7724 (external), and with the mean cumulative/dynamic Area Under the Curve (mean AUC) score of 0.747 (internal) and 0.763 (external). Moreover, the Integrated Brier Score (IBS) of the NMTLR model was 0.062 (internal) and 0.043 (external). The SHAP analysis showed over one-third of the top 20 features in the NMTLR model were comorbidities and medications, including a newly relevant drug named psychoanaleptics. Furthermore, the compact NMTLR model also performed well.

**Conclusion:**

Despite the limited representation of modern HF medications, both the full and compact NMTLR models are useful for predicting HF readmission. Additionally, psychoanaleptics might be a new predictor of readmission, warranting increased clinical attention.

## Highlights

• First study to develop a model using total comorbidities and medication data.
• Fill the gap in the readmission rate of critically ill HF pts in the 90-day vulnerable phase.
• Model results suggest psychoanaleptics may be linked to HF readmissions.
• Construct full & compact models with good performance for diverse clinical settings.

## Introduction

Heart Failure (HF) is a leading cause of hospitalization worldwide,^1^ posing a significant challenge to human health and affecting approximately 40 million patients.^2^^,^^3^ Studies indicated that the readmission rates for HF remained high,^4^ and the associated treatment costs were substantial,^5^^,^^6^ especially among critically ill HF patients admitted to the Intensive Care Unit (ICU).^7^^,^^8^ Therefore, developing a predictive model to identify critically ill HF patients at high risk of readmission is an urgent priority.

Existing HF models predominantly focused on predicting 30-day readmission risk.^9^, ^10^, ^11^, ^12^, ^13^ Only a minority of these models extended their predictions to cover the 90-day period, and their overall predictive performance fell short of the desired standard.^9^^,^^14^, ^15^, ^16^ However, the vulnerable phase of HF typically extended up to 90 days rather than the brief 30-day window.^17^ Utilizing a 90-day timeframe might provide a more comprehensive reflection of the readmission scenario during the vulnerable phase.^18^^,^^19^ Consequently, an optimized predictive tool that precisely forecasts the readmission risk of critically ill HF patients throughout this extended 90-day vulnerable phase still needs to be devised.

Moreover, existing 90-day readmission models for HF predominantly relied on routine fundamental information, basic comorbidities, and a limited set of medications.^9^^,^^14^, ^15^, ^16^ Early research indicated that a substantial proportion of HF patients contended with multiple comorbidities and managed at least 10 medications, which increased the likelihood of adverse drug events and might lead to an increased risk of readmission for HF patients.^20^ Meanwhile, studies showed that comorbidities significantly affected the prognosis of HF, and medications also played a crucial role in HF management.^21^^,^^22^ Therefore, a more comprehensive inclusion of these factors might improve prediction accuracy.

Based on the above background, the authors plan to utilize the open-access Medical Information Mart for Intensive Care III database version 1.4 (MIMIC-III v1.4) and MIMIC-IV v2.2 to construct a predictive model. This model would be grounded in a more comprehensive assessment of comorbidities and medications, aiming to accurately predict the 90-day readmission risk of critically ill HF patients. Furthermore, the authors attempt to utilize the Shapley Additive exPlanations (SHAP) method to interpret the model and delve into the factors influencing HF readmission. Through these efforts, the authors aspire to furnish more pragmatic predictive tools for the effective management of readmission in critically ill HF patients.

## Material and methods

### Data source

The present data was obtained from the MIMIC databases (MIMIC-III v1.4 and MIMIC-IV v2.2), which were publicly available retrospective clinical databases.^23^^,^^24^ The MIMIC databases were approved by the Massachusetts Institute of Technology Institutional Review Board (MIT-IRB), and the health information within the databases was de-identified, thus eliminating the necessity for patient-informed consent.^23^^,^^24^ The authors successfully completed the Collaborative Institutional Training Initiative (CITI) program course, obtaining certification to extract data from the databases for research purposes (certificate numbers: 56318333 and 56998638). This retrospective cohort study followed the STROBE statement.

### Study population

The authors considered all patients in the MIMIC databases, applying specific inclusion and exclusion criteria. The inclusion criteria were defined as follows: 1) Patients diagnosed with HF based on the International Classification of Diseases (ICD), 9th and 10th edition codes; 2) The diagnostic sequence for HF was in the top three; 3) Patients admitted to the ICU; and 4) Individuals aged 18 years or older. The exclusion criteria encompassed patients who experienced in-hospital mortality or died directly without subsequent hospital readmission.

### Data extraction and preprocessing

The anticipated outcome of this study was the probability of readmission due to HF within 90 days following discharge. The authors defined readmission due to HF based on the top three HF diagnostic sequence numbers, which were a criterion intended to mitigate confounding factors. Drawing inspiration from prior HF readmission studies,^14^^,^^25^, ^26^, ^27^ the authors identified variables for analysis, including demographics and vital signs. In cases where multiple recorded outcomes were present, the authors selected the first documented value.

Concerning the extraction of comorbidities and medications, the authors collected all data for patients. However, the authors excluded non-disease diagnoses (e.g., accidents), topical medications, and medications categorized under V. Next, the authors retained data with a contribution rate of 80 % or more to disease diagnoses and medications by calculating cumulative shares. Subsequently, the present data was subcategorized according to the 10th edition of the ICD and the ATC/DDD index for 2023. Notably, the exposure duration of medications was determined by calculating the start and end dates, with repeated use of similar drug classes on the same day not being cumulatively counted.

Variables with missing data, a common occurrence in the MIMIC databases, were addressed by excluding those with missing values exceeding 30 % or employing multiple imputation to impute features with missing data <30 %.^28^ The detailed missing values of the data were shown in Table S1. To enhance data reliability and eliminate dimensionality, normalization of indicators was deemed necessary. The selected formula $x*=\frac{x-\text{min}*0.99}{\text{max}-\text{min}}$ prevented zero minimum values in partial continuous variables (e.g., age, Body Mass Index [BMI], and Length Of Stay [LOS]).^29^ For the exposure duration of drugs, a specific formula $x*=\frac{x}{\text{length}\;\text{of}\;\text{hospital}\;\text{stay}}$ was applied. Additionally, variables with high correlation were eliminated through correlation analysis.

### Model development and validation

Utilizing a computer-generated sequence of random numbers, the authors randomly selected 80 % of the samples from MIMIC-VI as the development set and the remaining 20 % as the validation set. Furthermore, the authors utilized samples from MIMIC-III as an independent test set to further assess the applicability of the developed models. For the development of predictive models, the authors chose three machine learning models: two based on neural networks (Deep Learning Survival [DeepSurv] and Neural Multitasking Logistic Regression model [NMTLR]) and one employing ensemble learning (Random Survival Forest model [RSF]). Meanwhile, the authors constructed a multivariate Cox proportional hazards (CoxPH) model for comparative analysis. Hyperparameter tuning was performed for the three machine learning models using a randomized search method, conducting 100 experiments for each model and ultimately selecting the parameter set with the highest Concordance index (C-index).

Model performance was evaluated using the C-index and the mean cumulative/dynamic Area Under the Curve (mean AUC) score. To evaluate the time-dependent sensitivity and specificity of the model, a Receiver Operating Characteristic (ROC) curve was generated, and the Area Under the Curve (AUC) value was calculated for 90 days. The Integrated Brier Score (IBS) was also calculated to determine the models' overall performance across all available periods.^30^ A lower score indicates better calibration, and only models with scores below 0.25 are deemed useful in practice. Additionally, Decision Curve Analysis (DCA) was conducted to assess the decision models' utility by quantifying the net benefit at different threshold probabilities.

### Machine learning explainable tool

The interpretability of the predictive model was assessed using permutation importance and SHAP analyses. Permutation importance is determined by measuring the increase in the prediction error of models after randomly shuffling each feature. Meanwhile, SHAP is also a versatile method that enables the precise calculation of the contribution and impact of each feature on the final prediction. The SHAP values indicate the extent to which each predictor positively or negatively influences the target variable.

In order to be more practical in clinical settings, the authors selected 20 predictive factors with the highest SHAP importance to establish an accurate and compact model.

### Statistical analysis

Depending on whether continuous variables fit into a normal distribution or not, they were expressed as mean and Standard Deviation (SD) values or median and Interquartile Range (IQR) values. Frequency and percentage figures were used to summarize categorical variables; p-values were regarded as statistically significant if they were <0.05. Data preprocessing was done using R (version 4.3.2). Python (3.7) was used to implement the models.

## Results

### Patient characteristics

**Fig. 1:**
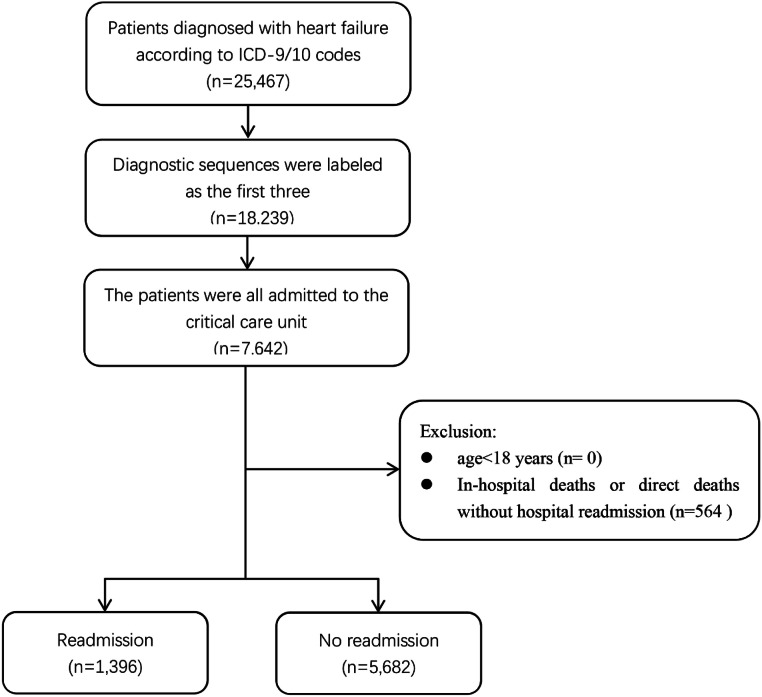
Flowchart of study inclusion.

The patient screening flowchart is depicted in Fig. 1. Out of the 25,467 patients with HF in the MIMIC-IV dataset, a total of 7078 adult patients diagnosed with HF met the inclusion and exclusion criteria and were included in the final cohort for this study. The 75 baseline clinical characteristics between the readmission group within 90 days after discharge and the non-readmission group were listed in Table 1. Both groups of patients were elderly, and the patients in the readmission group were older than those in the non-readmission group. In terms of comorbidities, more than half of the patients in both groups had hypertensive diseases, metabolic disorders, renal failure, and ischaemic heart diseases. Additionally, regarding medication during hospitalization, the drugs with longer average days of use in both groups primarily included platelet aggregation inhibitors, diuretics, beta-blockers, lipid-modifying agents, and analgesics. The days of use of these drugs were longer in the readmission group than in the non-readmission group. The drug usage of the participants from both MIMIC III and MIMIC IV in this study is listed in Supplementary Table S2.

**Table 1:** Baseline characteristics of participants with heart failure (*n* = 7078).

Characteristic	No readmission	Readmission	p-value
	*n* = 5682	*n* = 1396	
***Demographics***			
Age, years	74.0 (64.0‒83.0)	75.0 (65.0‒84.0)	0.036
Sex, male, n ( %)	3110 (54.7)	732 (52.4)	0.13
Ethnicity, n ( %)			<0.001
White	4026 (70.9)	1027 (73.6)	
Hispanic	158 (2.8)	58 (4.2)	
Other	226 (4.0)	62 (4.4)	
Black	592 (10.4)	190 (13.6)	
Unknown	541 (9.5)	19 (1.4)	
Asian	126 (2.2)	34 (2.4)	
American Indian	13 (0.2)	6 (0.4)	
Marital_status, n ( %)			<0.001
Married	2557 (45.0)	647 (46.3)	
Single	1245 (21.9)	331 (23.7)	
Widowed	1172 (20.6)	301 (21.6)	
Divorced	401 (7.1)	105 (7.5)	
Other	307 (5.4)	12 (0.9)	
***APS III***	41.0 (33.0‒52.0)	44.0 (35.0‒53.0)	<0.001
***SOFA***	4.0 (2.0‒6.0)	4.0 (2.0‒6.0)	0.791
***Vital signs***			
Height	168.0 (160.0‒175.9)	168.0 (157.0‒175.0)	0.023
Weight	80.2 (66.8‒96.1)	79.9 (66.8‒95.4)	0.604
BMI	28.5 (24.4‒33.4)	28.7 (24.5‒33.7)	0.412
HR	84.0 (73.0‒97.0)	84.0 (73.0‒97.0)	0.715
SBP_mean	115.2 (106.3‒127.4)	114.3 (105.3‒127.0)	0.099
DBP_mean	60.5 (54.0‒68.4)	60.2 (53.7‒68.1)	0.339
MBP_mean	75.8 (69.9‒82.9)	75.3 (69.3‒82.2)	0.021
MAP	78.6 (72.9‒86.5)	78.6 (72.5‒85.9)	0.228
RR_mean	18.0 (15.0‒23.0)	19.0 (16.0‒23.0)	0.023
SPO_2_	98.0 (95.0‒100.0)	98.0 (95.0‒100.0)	0.031
Temperature_mean	36.7 (36.5‒36.9)	36.7 (36.5‒36.9)	0.001
Urineoutput	1600.0 (980.5‒2480.0)	1575.0 (923.8‒2575.0)	0.318
***Laboratory findings (std)***			
Glucose_mean	130.0 (113.0‒156.7)	133.5 (113.1‒166.8)	0.004
Aniongap	14.0 (12.0‒17.0)	15.0 (13.0‒17.0)	0.01
Bicarbonate	25.0 (22.0‒28.0)	25.0 (22.0‒28.0)	0.719
BUN	24.0 (17.0‒38.0)	29.0 (20.0‒45.2)	<0.001
Creatinine	1.1 (0.9‒1.6)	1.3 (1.0‒1.9)	<0.001
Sodium	139.0 (136.0‒141.0)	139.0 (136.0‒141.0)	<0.001
Potassium	4.2 (3.8‒4.6)	4.2 (3.8‒4.6)	0.153
Hemoglobin	11.0 (9.4‒12.6)	10.5 (9.0‒12.0)	<0.001
MCH	29.8 (28.2‒31.4)	29.7 (27.8‒31.2)	0.002
MCV	91.0 (87.0‒95.0)	91.0 (87.0‒95.0)	0.322
MCHC	32.6 (31.5‒33.7)	32.4 (31.3‒33.5)	<0.001
Platelet	204.0 (158.0‒260.0)	204.0 (161.0‒262.0)	0.4
RBC	3.8 (3.2‒4.3)	3.6 (3.1‒4.1)	<0.001
RDW	14.7 (13.7‒16.2)	15.3 (14.2‒16.8)	<0.001
WBC	9.0 (6.8‒12.2)	8.7 (6.6‒12.0)	0.034
INR	1.3 (1.1‒1.5)	1.3 (1.1‒1.8)	<0.001
PT	13.9 (12.3‒16.9)	14.6 (12.6‒19.3)	<0.001
PTT	31.7 (27.9‒39.8)	32.2 (28.2‒39.4)	0.168
***CRRT, n ( %)***	112 (2.0)	29 (2.1)	0.883
***Ventilation, n ( %)***	4863 (85.6)	1183 (84.7)	0.448
***Comorbidity, n ( %)***			
Ischaemic heart diseases	3351 (59.0)	883 (63.3)	0.004
Metabolic disorders	4325 (76.1)	1070 (76.6)	0.703
Renal failure	2867 (50.5)	909 (65.1)	<0.001
Hypertensive diseases	4381 (77.1)	1126 (80.7)	0.005
Chronic rheumatic heart diseases	1444 (25.4)	393 (28.2)	0.04
Diabetes mellitus	2274 (40.0)	661 (47.3)	<0.001
Chronic lower respiratory diseases	1643 (28.9)	431 (30.9)	0.159
Influenza and pneumonia	730 (12.8)	189 (13.5)	0.52
Nutritional anaemias	1341 (23.6)	349 (25.0)	0.288
Pulmonary heart disease and diseases of pulmonary circulation	1106 (19.5)	321 (23.0)	0.004
***Duration of drugs use, days***			
H2RA	2.1 ± 4.0	1.9 ± 4.2	0.105
PPI	4.1 ± 6.5	5.0 ± 7.8	<0.001
Antiemetics and antinauseants	2.5 ± 5.0	2.8 ± 6.3	0.133
Insulins and analogues	4.3 ± 6.3	5.0 ± 7.2	<0.001
Vitamins	3.2 ± 5.9	4.0 ± 7.6	<0.001
Vitamin K antagonists	2.0 ± 3.9	2.5 ± 5.3	<0.001
Heparin	2.0 ± 4.6	2.3 ± 5.1	0.01
Platelet aggregation inhibitors	5.9 ± 6.4	7.0 ± 8.5	<0.001
Antianemic preparations	1.9 ± 4.7	2.5 ± 6.3	<0.001
Antiarrhythmics	1.7 ± 4.2	2.0 ± 4.7	0.069
Vasodilators	2.1 ± 3.7	2.3 ± 4.0	0.045
Diuretics	6.1 ± 6.0	7.1 ± 7.3	<0.001
Vasoprotectives	5.7 ± 5.9	6.1 ± 6.2	0.005
Beta-blockers	6.1 ± 6.0	6.7 ± 6.6	0.001
ACEI	1.8 ± 3.3	2.0 ± 3.7	0.1
Lipid-modifying agents	5.8 ± 6.4	6.9 ± 8.6	<0.001
Thyroid therapy	1.6 ± 4.5	1.9 ± 4.7	0.071
Pancreatic hormones	4.4 ± 6.4	4.9 ± 7.1	0.003
Antibacterials	4.2 ± 5.3	4.4 ± 6.2	0.175
Analgesics	7.4 ± 7.0	8.1 ± 9.0	0.003
Psycholeptics	3.5 ± 5.8	4.1 ± 7.2	0.003
Psychoanaleptics	2.9 ± 5.3	3.7 ± 6.6	<0.001
Drugs for obstructive airway diseases	4.4 ± 6.6	5.2 ± 7.9	<0.001
**First_hosp_stay, n ( %)**	4834 (85.1)	1127 (80.7)	<0.001
**Hospstay_seq**	1.0 (1.0‒1.0)	1.0 (1.0‒1.0)	<0.001
**Length of hospital stay, days**	2.1 ± 4.0	1.9 ± 4.2	0.865
**Length of ICU stay, days**	4.1 ± 6.5	5.0 ± 7.8	<0.001

APS III, Acute Physiology Score III; SOFA, Sequential Organ Failure Assessment score; BMI, Body Mass Index; HR, Heart Rate; SBP, Systolic Blood Pressure; DBP, Diastolic Blood Pressure; MBP, Mean Blood Pressure; MAP, Mean Artery Pressure; RR, Respiratory Rate; SPO_2_, Pulse Oxygen Saturation; BUN, Blood Urea Nitrogen; MCH, Mean Corpuscular Hemoglobin; mcv, mean corpuscular volume; MCHC, Mean Corpuscular Hemoglobin Concentration; RBC, Red Blood Cell; RDW, Red Blood Cell Distribution Width; WBC, White Blood Cell; INR, International Normalized Ratio; PT, Prothrombin Time; PTT, Partial Thromboplastin Time; CRRT, Continuous Renal Replacement Therapy; H2RAs, Histamine H2 Receptor Antagonists; PPI, Proton Pump Inhibitor; ACEI, Angiotensin-Converting Enzyme Inhibitor.

### Model building and evaluation

After analyzing variable correlations using the Spearman test, a correlation heat map was generated (Fig. S1). Variables with correlations exceeding 0.6 were excluded, while certain variables with potential impact on the outcome (e.g., the number of previous HF hospitalizations a patient (hospstay_seq)) were retained.^17^ Then, the remaining 67 variables were used for modeling.

**Fig. 2:**
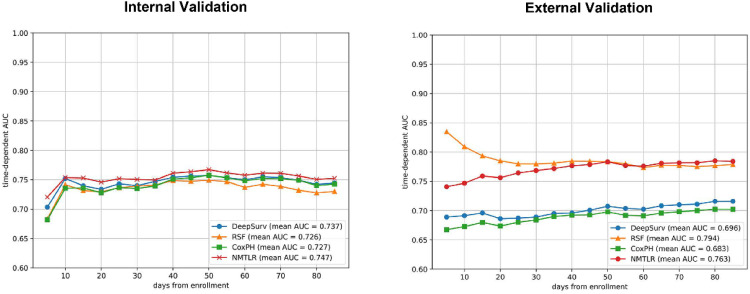
Time-dependent Area Under the Curve (AUC) for CoxPH, RSF, DeepSurv and NMTLR models.

**Fig. 3:**
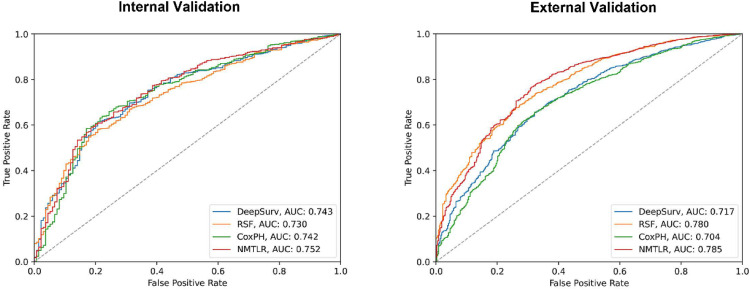
The Receiver Operating Curves (ROC) of 90 day readmission predictions for CoxPH, RSF, DeepSurv and NMTLR models.

In the training dataset, CoxPH, DeepSurv, RSF, and NMTLR models were established. The NMTLR model outperformed the other three models in terms of the C-index (C-index in internal validation cohort: CoxPH: 0.7300, DeepSurv: 0.7322, RSF: 0.7225, NMTLR: 0.7408; external validation cohort: CoxPH: 0.6950, DeepSurv: 0.7067, RSF: 0.7694, NMTLR: 0.7724). Fig. 2 shows the time-dependent average AUCs for the four models. In both internal and external validation, the time-dependent AUCs of the NMTLR model exceeded 0.7 in predicting both long-term and short-term readmission states. The ROCs for predicting 90-day readmission of the four models were shown in Fig. 3. Notably, the NMTLR model almost exhibited the highest predictive performance in both internal and external validation.

To further evaluate the models, the IBSs were calculated and presented in Supplementary Material Fig. S2. The corresponding prediction error curves of each model's BS in internal and external validation over time were all <0.25, indicating good discriminative ability and reliability of these models.

**Fig. 4:**
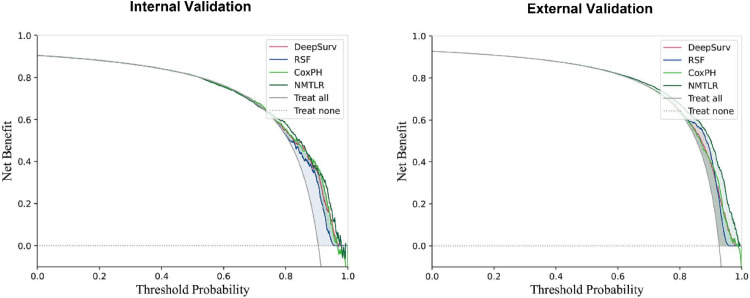
Decision curve analysis for CoxPH, RSF, DeepSurv, and NMTLR models. The X-axis indicates the threshold probability for the critical care outcome and Y-axis indicates the net benefit. The solid gray line represents the net benefit when all patients are treated; the dashed gray line (at 0 on the Y-axis) represents the net benefit when all patients are not treated.

The DCA plots (Fig. 4) illustrated the net benefit of the four models at varying threshold probabilities. The treatment strategy curve provided by any of the models was above both the treat-all and treat-none curves, indicating that all four models outperformed the default strategy of either treating all patients or not treating patients. Furthermore, in both internal and external validation, the NMTLR model outperformed the other machine learning models in terms of net benefit under most threshold probabilities.

### Feature importance

The assessment of variable permutation importance revealed features crucial for the prediction accuracy of models (refer to Fig. S3). The feature importance rankings based on this method for the four models were detailed in Supplementary Table S3.

**Fig. 5:**
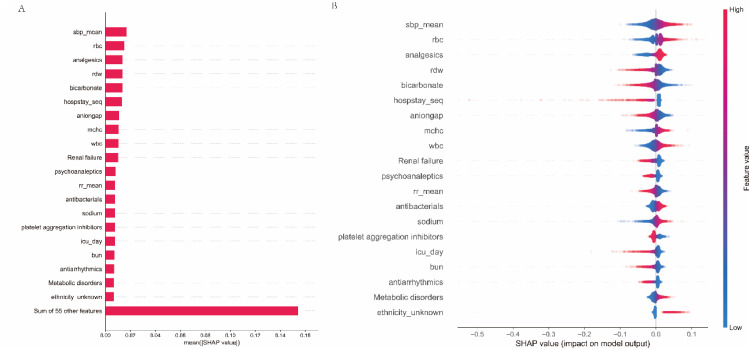
Interpreting the results of NMTLR model using SHAP explainer. Bar plots of mean absolute SHAP values: ranking of feature importance indicated by SHAP (A). The matrix plot depicts the importance of each covariate in the development of the final predictive model. SHAP summary plots for the top 20 clinical features (B): The higher the SHAP value of a feature, the higher the probability of no readmission development. Each line represents a feature, and the abscissa is the SHAP value. Red dots represent higher feature values, and blue dots represent lower feature values.

Moreover, the SHAP algorithm was used to obtain the importance of each predictor variable to the outcome predicted by the NMTLR model. The most influential variables were presented in descending order on the Bar plots of mean absolute SHAP values (see Fig. 5). Among the top 20 predictive variables, comorbidities and medications included analgesics, renal failure, psychoanaleptics, antibacterials, platelet aggregation inhibitors, antiarrhythmics, and metabolic disorders, with medications accounting for a quarter of the top 20 variables. Additionally, the authors used SHAP summary plots (Fig. 5) to visualize readmission risk factors, which revealed not only the relative importance of features but also their actual relationship with predicted outcomes. Notably, analgesics, antibacterials, and metabolic disorders were protective factors for the non-readmission outcome in HF patients. The longer the duration of analgesics and antibacterials use in the hospital, the higher the probability that the patient will not be readmitted, thus lowering the readmission probability. However, renal failure as well as increased days of use of psychoanaleptics, platelet aggregation inhibitors, and antiarrhythmics had the opposite effect.

### Performance of the compact NMTLR models

The compact NMTLR model was developed based on the top 20 predictive factors selected according to the SHAP values of the optimal NMTLR model. Compared with the full model performance of 67 variables, the compact NMTLR model exhibited slightly lower C-indexes (internal: 0.7390, external: 0.7155), mean AUCs (see Fig. S4), AUCs (Fig. S5), and IBSs (Fig. S6). The DCA diagram of the compact model exhibited appreciable net benefits (Fig. S7). However, in terms of clinical utility, the compact NMTLR model was considered more practical in clinical settings.

## Discussion

As far as the authors know, this is the first clinical prediction model developed and validated to assess the readmission rate of critically ill HF patients within the 90-day vulnerable phase, based on relatively comprehensive clinical comorbidities and medication data. The results indicated that all four algorithms had satisfactory predictive performance, with the NMTLR model notably outperforming the other three. Additionally, both the full NMTLR model, including 67 predictors and the compact NMTLR model containing only 20 predictors demonstrated good model performance in internal and external validations, which provided strong evidence for the robustness of the present model. Overall, this study innovatively explored a more comprehensive set of clinical comorbidities and medication regimens to predict the prognosis of critically ill HF patients during the vulnerable phase, which might assist physicians in assessing the readmission probability of such patients and determining further treatment programs.

The vulnerable phase is a critical phase for HF, during which the majority of readmission events occur.^17^ Moreover, critically ill HF inpatients are frequently admitted to the ICU,^8^ especially those with multiple complex comorbidities, whose inherent frailty and comorbidities significantly increase the risk of readmissions after discharge.^17^ Furthermore, patients with multiple comorbidities often require additional medications, which might lead to drug interactions that further complicate the already complex HF treatment regimens.^31^ Studies indicated that the complexity of medication regimens was a key factor in adverse drug events,^31^ potentially exacerbating HF and increasing the risk of readmission. Therefore, it is reasonable and feasible to use the comorbidities and medication exposures in this population as indicators for HF patient prognosis assessment, as they significantly impact the occurrence of readmission events. Additionally, the full model and compact model included more extensive predictive indicators compared to models from previous studies, ultimately encompassing more than just conventional HF comorbidities and treatment medications. Therefore, the present model might provide new insights for predicting readmission risks for HF and other diseases.

Deep learning, due to its superior modeling capabilities and predictive performance, was widely used in constructing clinical prediction models.^32^^,^^33^ Currently, this approach has been refined by integrating time variation with deep learning to develop models that predicted event probabilities over time.^30^ Such deep learning models significantly outperformed others in handling large samples, multivariate, and nonlinear data.^30^ In this context, the authors constructed two deep learning models (DeepSurv and NMTLR) to predict the readmission rates of critically ill HF patients and compared their performance with two classical models (CoxPH and RSF). The NMTLR model exhibited optimal performance in both internal and external validations. Unlike traditional prediction models that could only predict binary outcomes (readmitted or not), the NMTLR model was more flexible and could directly predict the probability function of patients not being readmitted, thus obtaining readmission probabilities at any time point. However, the full model containing 67 variables had poor operability in everyday clinical settings. Therefore, the authors recommended its application in large hospitals equipped with advanced diagnostic facilities and abundant medical resources. Additionally, the authors developed a compact NMTLR model including 20 selected predictive factors, retaining similar discriminative power and accuracy but with greater operability, which was recommended for use in routine clinical practice. Future software programming work embedding these models into clinical workflows to achieve real-time interactive integration between Electronic Health Record (EHR) systems and predictive models and to synchronously develop clinical decision support tools based on dynamic risk assessment would be greatly appreciated.

In this study, the authors primarily used the SHAP method to interpret the NMTLR model and identified key variables associated with non-readmission in critically ill HF patients. More than one-third of the top 20 variables were comorbidities and medications, which further supported the validity of these insights. Significant comorbidities associated with outcomes included renal failure and metabolic disorders, where renal failure was a risk factor for non-readmission in HF patients, possibly due to worsening HF from renal insufficiency.^34^ However, metabolic disorders were found to be protective factors for outcomes, which was inconsistent with previous research findings.^35^^,^^36^ Although the relevance and importance shown by the interpretability results do not guarantee biological significance,^37^ these results are still worth paying certain attention to, and the underlying mechanisms might require further investigations. Additionally, the present analysis also identified five key drug categories. Notably, the authors found that psychoanaleptics unrelated to HF treatment had a significant impact on the present model. The increased frequency of psychoanaleptics use might raise readmission risks, whereas previous models did not include this indicator.^9^^,^^14^, ^15^, ^16^ Related studies showed that even at standard doses, psychoanaleptics could cause cardiovascular adverse events.^38^ Therefore, the stringent and interpretable results produced by the NMTLR model not only enhanced its credibility but also provided valuable predictive factors and a theoretical basis for future readmission studies.

An interesting point is that multiple early studies showed that the persistent hemodynamic congestion state present at the time of patient discharge is a key factor influencing high mortality and rehospitalization rates during the vulnerable phase.^17^^,^^39^^,^^40^ Although congestion is often accompanied by relatively obvious clinical signs such as peripheral edema, lung rales, and jugular venous distension, the immediate identification and accurate assessment of these signs remain a major challenge for doctors in busy clinical practice.^39^ Fortunately, recent research unveiled new potential directions for us, namely assessing the causes of congestion in HF by monitoring Brain Natriuretic Peptide (BNP) concentrations, estimating Plasma Volume Status (ePVS), utilizing Bioimpedance Vector Analysis (BIVA) technology, and evaluating the Blood Urea Nitrogen to Creatinine Ratio (BUN/Cr), which represent different pathophysiological processes (hemodynamics, intravascular, and interstitial fluid retention) involved in congestion.^40^ Despite the historical limitations of the MIMIC databases, which did not include complete data on these relevant indicators (including BNP, ePVS, BIVA, and BUN/Cr), it is noteworthy that BUN was one of the top 20 variables in terms of importance and exhibited a relatively high weight in the present study. Considering that BUN is also an important indicator related to the congestion state,^40^ these findings indirectly suggest that this model eventually incorporates information related to the congestion state, although further supporting data are still needed.

This study inevitably had several limitations. First, the data were sourced from public databases, which had limited variables and might lack some critical predictive variables affecting readmission, such as regular post-discharge telephone calls.^17^ Second, the readmission information in the MIMIC databases was confined to a few institutions, lacking data on patients admitted to other facilities, which could introduce bias into the results. Third, the readmission prediction model was developed based on available inpatient treatment data, potentially overlooking crucial information from before admission and after discharge. Fourth, the early establishment of the databases meant the treatment data might not fully reflect current clinical practices. For instance, the representation of modern HF medications (e.g., angiotensin receptor-neprilysin inhibitor and sodium-glucose cotransporter 2 inhibitor) is limited, which restricts the generalizability of the findings to contemporary clinical settings. Additionally, although this model can provide potential clues for treatment regimens, proposing specific optimization strategies still requires further research and confirmation based on large-sample clinical data. These limitations should be addressed in future related studies.

## Conclusion

In summary, the authors developed and validated an interpretable full NMTLR model and an interpretable compact NMTLR model, both of which demonstrated good performance in predicting readmission risk for critically ill HF patients during the vulnerable phase. The interpretable results indicated that it was both reasonable and feasible to extract more comprehensive comorbidities and medication exposures as predictive indicators, potentially uncovering new predictors of HF readmission. Moreover, this model might assist physicians in identifying HF patients at high risk of readmission, enabling timely and appropriate treatment to reduce readmission rates and also offering new potential directions for exploring key indicators related to readmission in HF and other conditions.

## CRediT authorship contribution statement

**Meng-Han Jiang:** Conceptualization, Data curation, Formal analysis, Investigation, Methodology, Validation, Visualization, Writing – original draft, Writing – review & editing. **Fang Yu:** Data curation, Investigation, Writing – review & editing. **Hai-Ying Yang:** Conceptualization, Data curation, Methodology, Writing – original draft, Writing – review & editing. **Sun-Jun Yin:** Investigation, Writing – original draft, Writing – review & editing, Funding acquisition. **Li-Juan Yang:** Investigation, Writing – original draft, Writing – review & editing. **Yu Chen:** Methodology, Writing – original draft, Writing – review & editing. **De-Min Li:** Writing – original draft, Writing – review & editing. **Yu Guo:** Conceptualization, Formal analysis, Methodology. **Jia-De Zhu:** Investigation, Writing – original draft, Writing – review & editing. **Wen-Ke Cai:** Supervision, Resources, Writing – review & editing. **Gong-Hao He:** Conceptualization, Writing – review & editing, Project administration, Funding acquisition, Supervision.

## Conflicts of interest

The authors declare no conflicts of interest.

## Data Availability

The datasets generated and analyzed during the current study are available from the corresponding databases on reasonable request. The datasets generated and analyzed during the current study are available in the MIMIC databases (https://mimic.mit.edu/).

## Appendix Supplementary materials

Image, application 1
