# Supplementary material for: Predicting readmission rates in critically ill heart failure patients during a 90-day vulnerable phase using interpretable machine learning models
Source: Clinics (Sao Paulo). 2025 Sep 11;80:100775. doi: 10.1016/j.clinsp.2025.100775 (PMC12834062; doi:10.1016/j.clinsp.2025.100775)
Supplement: Supplementary file 1 [file mmc1.doc]

**List of Supplementary material**

**Table S1**: Missing number (%) for included variables in the MIMIC-IV and MIMIC-Ⅲ database.

**Table S2**: The drug usage situation of participants.

**Table S3**: Permutation feature importance for CoxPH, RSF, DeepSurv and NMTLR models.

**Fig. S1**: Heatmap of correlation coefficients between variables. They are represented by color depth, with a number closer to either end value implying a stronger negative correlation or positive correlation. Notes: *p ≤ 0.05; **p ≤ 0.01; ***p ≤ 0.001.

**Fig. S2**: Prediction error curves show the Brier score for CoxPH, RSF, DeepSurv and NMTLR models at each time point. As a benchmark, a useful model will have a Brier score below 0.25.

**Fig. S3**: Heatmap of feature importance for CoxPH, RSF, DeepSurv and NMTLR models. The values are expressed as a percentage reduction in the C-index after the value of a feature has been replaced by random numbers. Higher values suggest that a feature is more important in influencing the predictive accuracy of the corresponding models.

**Fig. S4**: Time-dependent area under the curve (AUC) for the compact NMTLR mode.

**Fig. S5**: The receiver operating curves (ROC) of 90-day readmission predictions for the compact NMTLR mode.

**Fig. S6**: Prediction error curves show the Brier score for the compact NMTLR mode at each time point. As a benchmark, a useful model will have a Brier score below 0.25.

**Fig. S7**: Decision curve analysis for the compact NMTLR mode. X-axis indicates the threshold probability for critical care outcome and Y-axis indicates the net benefit. The solid gray line represents the net benefit when all patients are treated; the dashed gray line (at 0 on the y-axis) represents the net benefit when all patients are not treated.

Table S1: Missing number (%) for included variables in the MIMIC-IV and MIMIC-Ⅲ database.

| **Characteristics, n (%)** | **MIMIC-IV**  **N=7078** | **MIMIC-III**  **N=5048** |
| --- | --- | --- |
| height | 1104 (15.6) | 422 (8.36) |
| weight | 7 (0.10) | 94 (1.86) |
| hr | 1 (0.01) | 96 (1.90) |
| sbp_mean | 7 (0.10) | 96 (1.90) |
| dbp_mean | 7 (0.10) | 96 (1.90) |
| mbp_mean | 4 (0.05) | 99 (1.97) |
| rr_mean | 4 (0.05) | 99 (1.97) |
| spo2 | 1 (0.01) | 98 (1.94) |
| temperature_mean | 411 (5.80) | 289 (5.73) |
| glucose_mean | 687 (9.70) | 53 (1.05) |
| aniongap | 354 (5.00) | 198 (3.92) |
| bicarbonate | 272 (3.84) | 86 (1.70) |
| bun | 17 (0.23) | 67 (1.33) |
| creatinine | 16 (0.22) | 52 (1.03) |
| sodium | 322 (4.54) | 64 (1.27) |
| potassium | 264 (3.73) | 59 (1.17) |
| hemoglobin | 268 (3.79) | 4 (0.08) |
| mch | 286 (4.04) | 61 (1.21) |
| mcv | 284 (4.01) | 61 (1.21) |
| mchc | 286 (4.04) | 60 (1.19) |
| platelet | 175 (2.47) | 53 (1.05) |
| rbc | 283 (4.00) | 60 (1.21) |
| rdw | 283 (4.00) | 60 (1.21) |
| wbc | 276 (3.90) | 87 (1.72) |
| inr | 685 (9.68) | 502 (9.94) |
| pt | 685 (9.68) | 346 (6.85) |
| ptt | 675 (9.54) | 212 (4.20) |
| urineoutput | 262 (3.70) | 205 (4.06) |

Abbreviations: hr, heart rate; sbp, systolic blood pressure; dbp, diastolic blood pressure; mbp, mean blood pressure; rr, respiratory rate; spo2: pulse oxygen saturation; mch, mean corpuscular hemoglobin; mcv, mean corpuscular volume; mchc, mean corpuscular hemoglobin contentration; rbc, red blood cell; rdw, red blood cell distribution width; wbc, white blood cell; inr, international normalized ratio; pt, prothrombin time; ptt, partial thromboplastin time.

**Table S2**: The drug usage situation of participants.

| **Characteristics** | **MIMIC-IV**  **(Training/Internal validation%)**  **N=7078** | **MIMIC-III**  **(External validation %)**  **N=5048** | **P-value** |
| --- | --- | --- | --- |
| **Drug usage, n (%)** |  |  |  |
| H2RA | 2447 (34.6%) | 1877 (37.2%) | 0.003 |
| PPI | 3602 (50.9%) | 2944 (58.3%) | <0.001 |
| antiemetics and antinauseants | 2698 (38.1%) | 1278 (25.3%) | <0.001 |
| insulins and analogues | 4229 (59.7%) | 2549 (50.5%) | <0.001 |
| vitamins | 2927 (41.4%) | 1552 (30.7%) | <0.001 |
| vitamin k antagonists | 2491 (35.2%) | 1710 (33.9%) | 0.138 |
| heparin | 2497 (35.3%) | 1468 (29.1%) | <0.001 |
| platelet aggregation inhibitors | 5437 (76.8%) | 3716 (73.6%) | <0.001 |
| antianemic preparations | 1877 (26.5%) | 1216 (24.1%) | 0.003 |
| antiarrhythmics | 1983 (28.0%) | 1359 (26.9%) | 0.19 |
| vasodilators | 3207 (45.3%) | 2648 (52.5%) | <0.001 |
| diuretics | 6207 (87.7%) | 4036 (80.0%) | <0.001 |
| vasoprotectives | 5932 (83.8%) | 3892 (77.1%) | <0.001 |
| Beta-blockers | 6040 (85.3%) | 4100 (81.2%) | <0.001 |
| ACEI | 2821 (39.9%) | 2463 (48.8%) | <0.001 |
| lipid modifying agents | 5130 (72.5%) | 3204 (63.5%) | <0.001 |
| thyroid therapy | 1353 (19.1%) | 764 (15.1%) | <0.001 |
| pancreatic hormones | 987 (19.6%) | 3885 (54.9%) | <0.001 |
| antibacterials | 5151 (72.8%) | 3526 (69.8%) | <0.001 |
| analgesics | 6239 (88.1%) | 4174 (82.7%) | <0.001 |
| psycholeptics | 3949 (55.8%) | 2983 (59.1%) | <0.001 |
| psychoanaleptics | 2934 (41.5%) | 1728 (34.2%) | <0.001 |
| drugs for obstructive airway diseases | 3761 (53.1%) | 2472 (49.0%) | <0.001 |
| ARB | 550 (10.9%) | 947 (13.4%) | <0.001 |
| ARNI | 6 (0.1%) | 62 (0.9%) | <0.001 |
| SGLT2i | 2(0.03%) | 0(0%) | 0.633 |

Abbreviations: H2RAs, histamine H2 receptor antagonists; PPI, proton pump inhibitor; ACEI, angiotensin-converting enzyme inhibitor; ARB, Angiotensin II Receptor Blockers; ARNI, Angiotensin Receptor-Neprilysin Inhibitor; SGLT2i, Sodium-Glucose Cotransporter 2 Inhibitor.

Table S3: Permutation feature importance for CoxPH, RSF, DeepSurv and NMTLR models.

| **Variable** | **CoxPH** | **DeepSurv** | **RSF** | | **NMTLR** | **Average** | |
| --- | --- | --- | --- | --- | --- | --- | --- |
| age | -0.00152 | 0.00015 | | 0.000609 | 0.001716 | | 0.000238 |
| bmi | 6.78E-05 | -0.00389 | | -0.00021 | 0.013591 | | 0.00239 |
| apsiii | 0.000784 | -0.00149 | | 0.001668 | 0.002792 | | 0.000939 |
| sofa | -0.00161 | 0.001165 | | 0.000624 | 0.002614 | | 0.0007 |
| hr | 0.001108 | -0.00093 | | 0.001051 | 0.001225 | | 0.000613 |
| sbp_mean | 0.04916 | 0.015141 | | 0.005361 | 0.033896 | | 0.025889 |
| dbp_mean | -0.00173 | 0.001285 | | 0.001066 | 0.001508 | | 0.000533 |
| rr_mean | 0.001726 | 0.003847 | | 0.001104 | 0.0028 | | 0.002369 |
| spo2 | 0.001341 | 0.00662 | | 0.004112 | 0.01527 | | 0.006836 |
| temperature_mean | 0.000422 | -0.00193 | | 0.001188 | 0.009076 | | 0.002189 |
| glucose_mean | 0.000219 | 0.001067 | | 0.000152 | 0.00791 | | 0.002337 |
| urineoutput | -0.00027 | 0.001616 | | 0.003259 | 0.001545 | | 0.001537 |
| aniongap | 0.014078 | 0.004756 | | 0.002901 | 0.013599 | | 0.008834 |
| bicarbonate | 0.013844 | 0.00553 | | 0.004356 | 0.015032 | | 0.009691 |
| bun | -0.00779 | -0.01504 | | -0.00267 | 0.005815 | | -0.00492 |
| creatinine | 0.005539 | -0.00049 | | -0.01181 | 0.001062 | | -0.00142 |
| sodium | -0.00076 | 0.000676 | | 0.002703 | 0.000468 | | 0.000772 |
| potassium | 0.001017 | 0.001653 | | 0.000419 | -0.00022 | | 0.000717 |
| mcv | 0.001357 | 0.000466 | | 0.001553 | -0.00061 | | 0.000692 |
| mchc | 0.002562 | 0.002044 | | 0.002703 | 0.009803 | | 0.004278 |
| platelet | 0.000128 | -0.00101 | | 0.00035 | -0.00272 | | -0.00081 |
| rbc | 0.033221 | 0.008649 | | 0.004508 | 0.017609 | | 0.015997 |
| rdw | 0.015691 | 0.017117 | | 0.004957 | 0.028021 | | 0.016447 |
| wbc | 0.012593 | 0.016148 | | 0.000503 | 0.026001 | | 0.013811 |
| inr | 0.000158 | -0.00646 | | -0.00053 | -0.00471 | | -0.00288 |
| ptt | -0.001 | -0.00069 | | -0.00101 | 0.003973 | | 0.000319 |
| Ischaemic heart diseases | -0.00213 | -0.00439 | | 6.85E-05 | 0.00329 | | -0.00079 |
| Metabolic disorders | 0.012503 | 0.005267 | | 0.001569 | 0.006506 | | 0.006461 |
| Renal failure | 0.004137 | 0.005838 | | -0.00077 | 0.003832 | | 0.00326 |
| Hypertensive diseases | -0.00045 | -7.51E-05 | | 0.000122 | 0.00075 | | 8.62E-05 |
| Chronic rheumatic heart diseases | 0.00566 | 0.00538 | | -0.00069 | 0.001404 | | 0.002938 |
| Diabetes mellitus | -0.00025 | -0.00041 | | 0.000693 | 0.003223 | | 0.000814 |
| Chronic lower respiratory diseases | -0.00068 | 0.000248 | | 0.000274 | 0.004731 | | 0.001144 |
| Influenza and pneumonia | -0.00134 | -0.00013 | | 0.000175 | 0.002822 | | 0.000382 |
| Nutritional anaemias | -0.00128 | -0.00104 | | 0.000404 | 0.005585 | | 0.000916 |
| Pulmonary heart disease and diseases of pulmonary circulation | 0.000211 | 0.003126 | | 0.002071 | 0.002703 | | 0.002028 |
| H2RA | 0.001816 | 2.25E-05 | | 0.001713 | 0.003907 | | 0.001865 |
| PPI | -4.52E-05 | -0.00086 | | 0.000632 | 0.005904 | | 0.001409 |
| antiemetics and antinauseants | -0.00024 | 0.001029 | | 0.000434 | -0.00016 | | 0.000267 |
| insulins and analogues | 0.000497 | -0.00086 | | 5.33E-05 | 0.002139 | | 0.000458 |
| vitamins | -0.00142 | 3.76E-05 | | 0.000731 | 0.007917 | | 0.001815 |
| vitamin k antagonists | 0.003836 | 0.008656 | | 0.004295 | 0.005251 | | 0.005509 |

Table S3 continued

| **Variable** | **CoxPH** | **DeepSurv** | | **RSF** | **NMTLR** | | **Average** |
| --- | --- | --- | --- | --- | --- | --- | --- |
| heparin | 0.00043 | -0.00072 | | 0.000129 | 0.001448 | | 0.000321 |
| platelet aggregation inhibitors | 0.009993 | 0.011068 | | 0.001089 | 0.008905 | | 0.007764 |
| antianemic preparations | 0.000196 | 0.00414 | | 0 | 0.000824 | | 0.00129 |
| antiarrhythmics | -0.00139 | 0.001578 | | 0.001462 | 0.003335 | | 0.001247 |
| vasodilators | 0.000211 | -0.00045 | | -0.00039 | -0.0011 | | -0.00043 |
| diuretics | 0.011915 | 0.009535 | | 0.003556 | 0.010375 | | 0.008845 |
| vasoprotectives | -0.0016 | 0.000654 | | 0.001607 | -0.00212 | | -0.00036 |
| Beta-blockers | 0.001877 | -0.00067 | | -0.00022 | -0.00102 | | -9.48E-06 |
| ACEI | 0.000445 | -0.00137 | | -0.00102 | 0.002325 | | 9.53E-05 |
| lipid modifying agents | 0.004047 | 0.004508 | | 4.57E-05 | -0.00426 | | 0.001086 |
| thyroid therapy | -0.00113 | 0.003058 | | 0.000579 | 0.00208 | | 0.001146 |
| antibacterials | 0.003753 | 0.004711 | | 0.002094 | 0.007672 | | 0.004558 |
| analgesics | 0.016226 | 0.013285 | | -0.00069 | 0.007768 | | 0.009148 |
| psycholeptics | 0.000543 | 0.000466 | | 0.000335 | 0.002822 | | 0.001041 |
| psychoanaleptics | -0.00157 | 0.000496 | | 0.002764 | -0.0031 | | -0.00035 |
| drugs for obstructive airway diseases | 0.000188 | 0.003254 | | 0.001828 | 0.001277 | | 0.001637 |
| hospstay_seq | -0.002 | 0.00018 | | 0.025586 | 0.008615 | | 0.008094 |
| icu_day | 0.014116 | 0.016846 | | 0.017903 | 0.011616 | | 0.01512 |
| hos_day | -0.00036 | 0.000819 | | 0.000723 | -0.00602 | | -0.00121 |
| sex_male | 0.004846 | -0.00143 | | 3.05E-05 | -0.00276 | | 0.000173 |
| ethnicity_asian | 0.022134 | 0.000571 | | 0 | 0.002429 | | 0.006284 |
| ethnicity_black | 0.097039 | -0.00064 | | 0.000586 | 0.003208 | | 0.025049 |
| ethnicity_hispanic | 0.051557 | 0.000173 | | 0.000137 | -0.00183 | | 0.012508 |
| ethnicity_other | 0.032346 | 0.001007 | | -6.09E-05 | -0.0001 | | 0.008297 |
| ethnicity_unknown | 0.038911 | 0.005891 | | 0.003236 | 0.019666 | | 0.016926 |
| ethnicity_white | 0.235212 | 0.008844 | | -0.00035 | 0.004478 | | 0.062046 |
| marital_status_married | 0.000852 | 0.004178 | | -9.90E-05 | 0.001077 | | 0.001502 |
| marital_status_other | 0.002404 | 0.001413 | | -0.00017 | 0.008927 | | 0.003144 |
| marital_status_single | 1.51E-05 | -0.00129 | | 0.000487 | -0.0002 | | -0.00025 |
| marital_status_widowed | -7.54E-06 | 7.51E-06 | | 0.000594 | -0.00316 | | -0.00064 |
| ventilation_yes | -0.00029 | -0.00143 | | -0.00043 | -0.00649 | | -0.00216 |
| crrt_yes | 0.002133 | 0.000301 | | 5.33E-05 | 0.002421 | | 0.001227 |
| first_hosp_stay_yes | 0.001417 | 0.002765 | 0.001279 | | -8.17E-05 | 0.001345 | |

Abbreviations: APS III, acute physiology score III; SOFA, sequential organ failure assessment score; bmi, body mass index; hr, heart rate; sbp, systolic blood pressure; dbp, diastolic blood pressure; mbp, mean blood pressure; map, mean artery pressure; rr, respiratory rate; spo2: pulse oxygen saturation; mch, mean corpuscular hemoglobin; mcv, mean corpuscular volume; mchc, mean corpuscular hemoglobin contentration; rbc, red blood cell; rdw, red blood cell distribution width; wbc, white blood cell; inr, international normalized ratio; pt, prothrombin time; ptt, partial thromboplastin time; CRRT, continuous renal replacement therapy; H2RAs, histamine H2 receptor antagonists; PPI, proton pump inhibitor; ACEI, angiotensin-converting enzyme inhibitor.


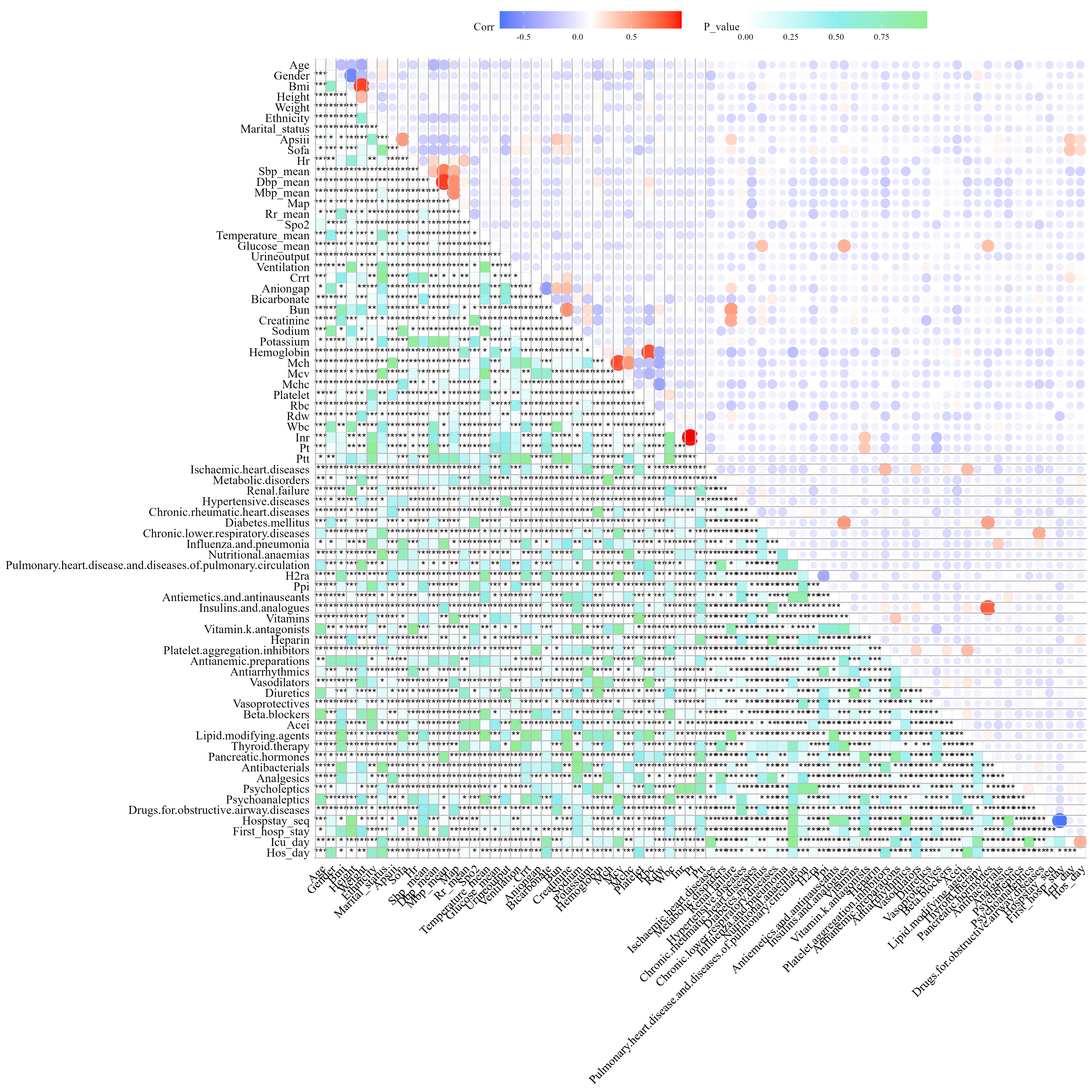


**Fig. S1**: Heatmap of correlation coefficients between variables. They are represented by color depth, with a number closer to either end value implying a stronger negative correlation or positive correlation. Notes: *p ≤ 0.05; **p ≤ 0.01; ***p ≤ 0.001.


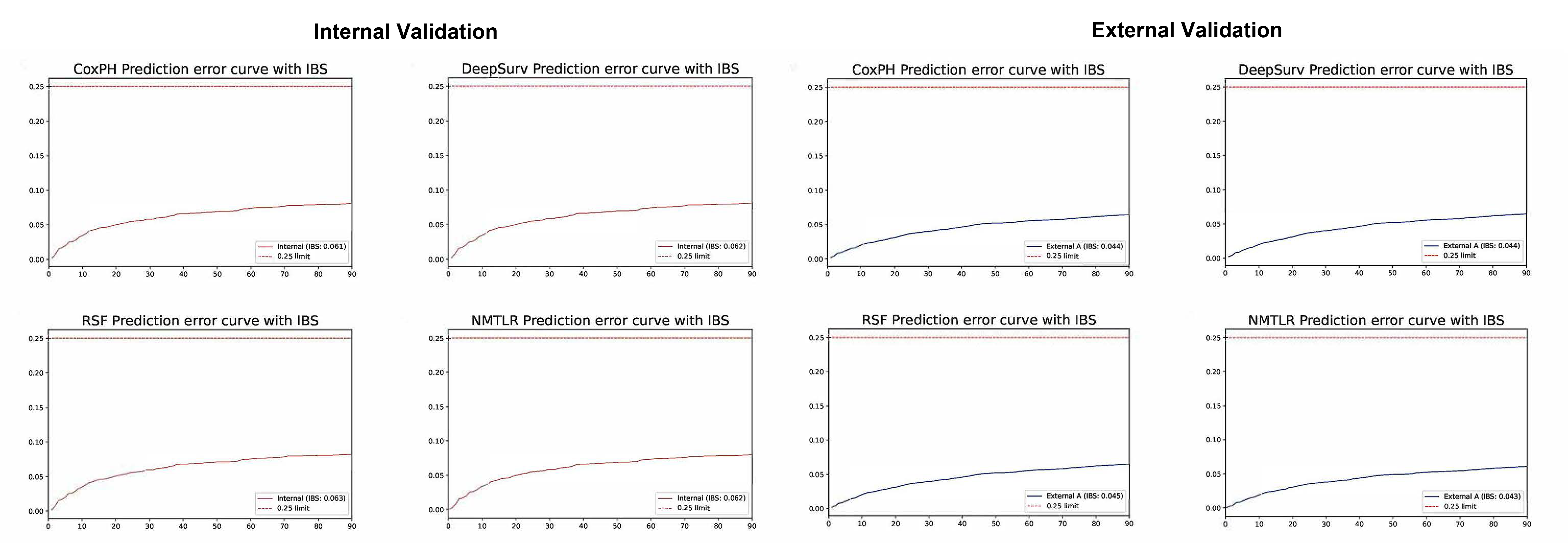


**Fig. S2**: Prediction error curves show the Brier score for CoxPH, RSF, DeepSurv and NMTLR models at each time point. As a benchmark, a useful model will have a Brier score below 0.25.


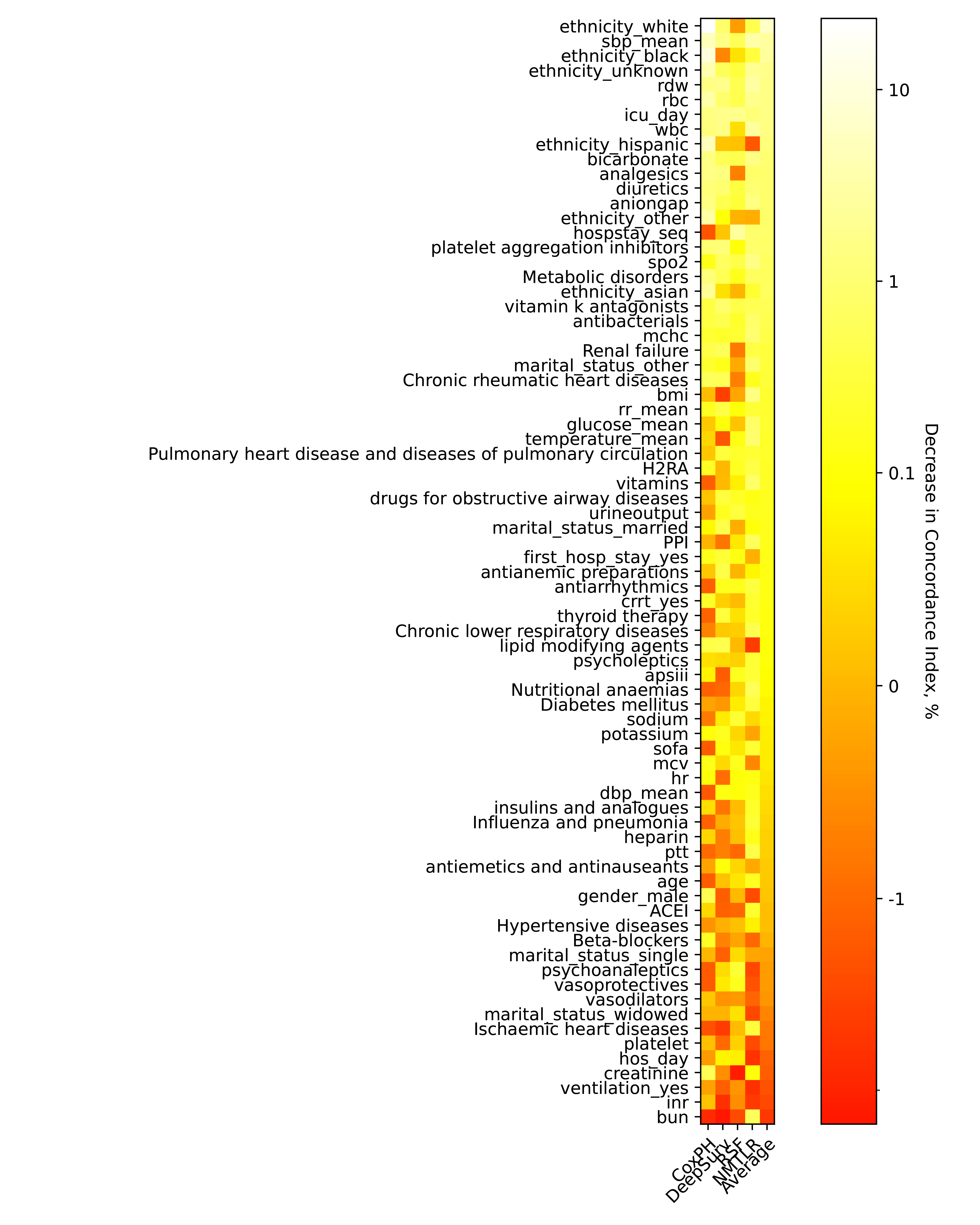


**Fig. S3**: Heatmap of feature importance for CoxPH, RSF, DeepSurv and NMTLR models. The values are expressed as a percentage reduction in the C-index after the value of a feature has been replaced by random numbers. Higher values suggest that a feature is more important in influencing the predictive accuracy of the corresponding models.


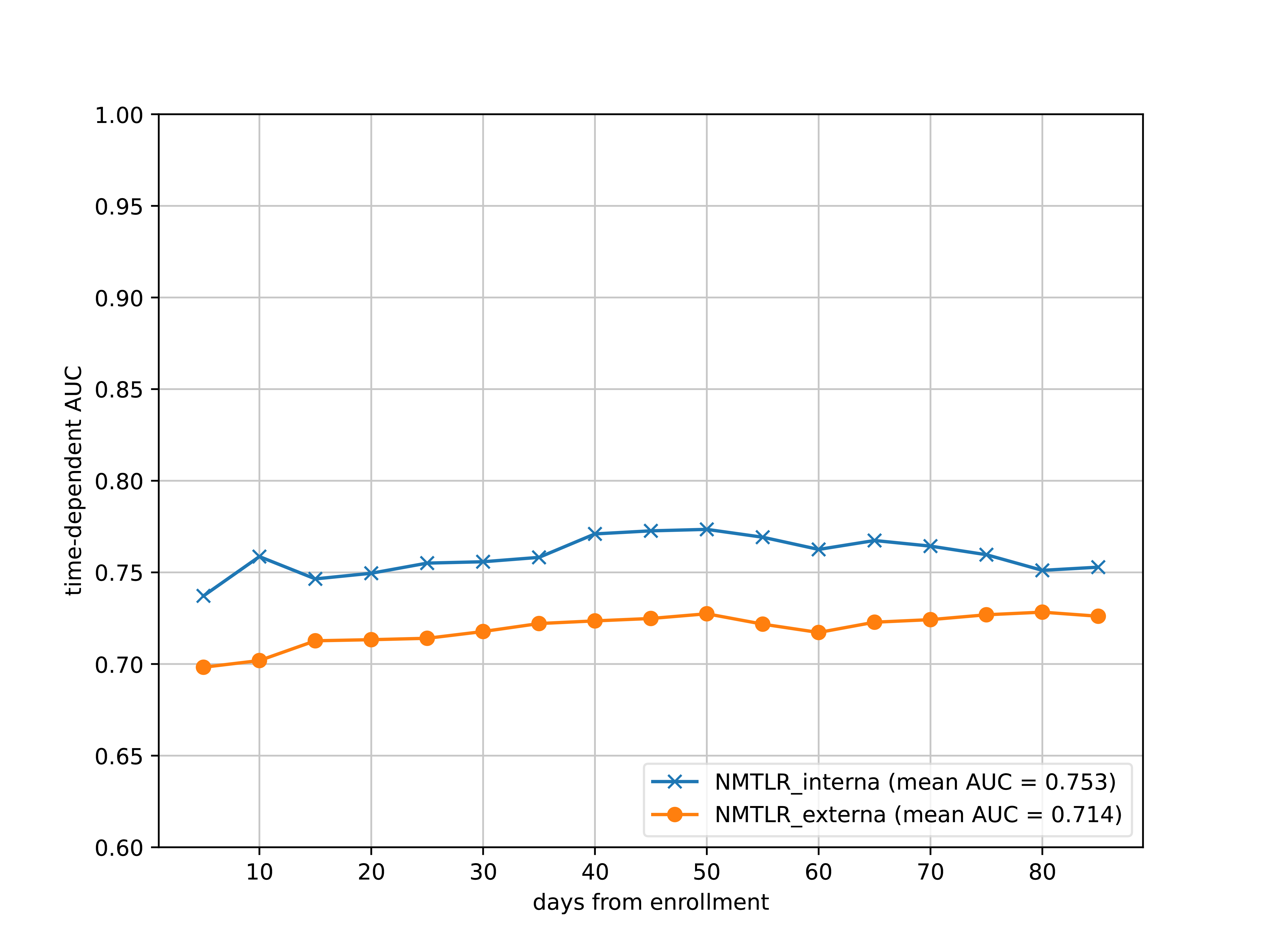


**Fig. S4**: Time-dependent area under the curve (AUC) for the compact NMTLR mode.


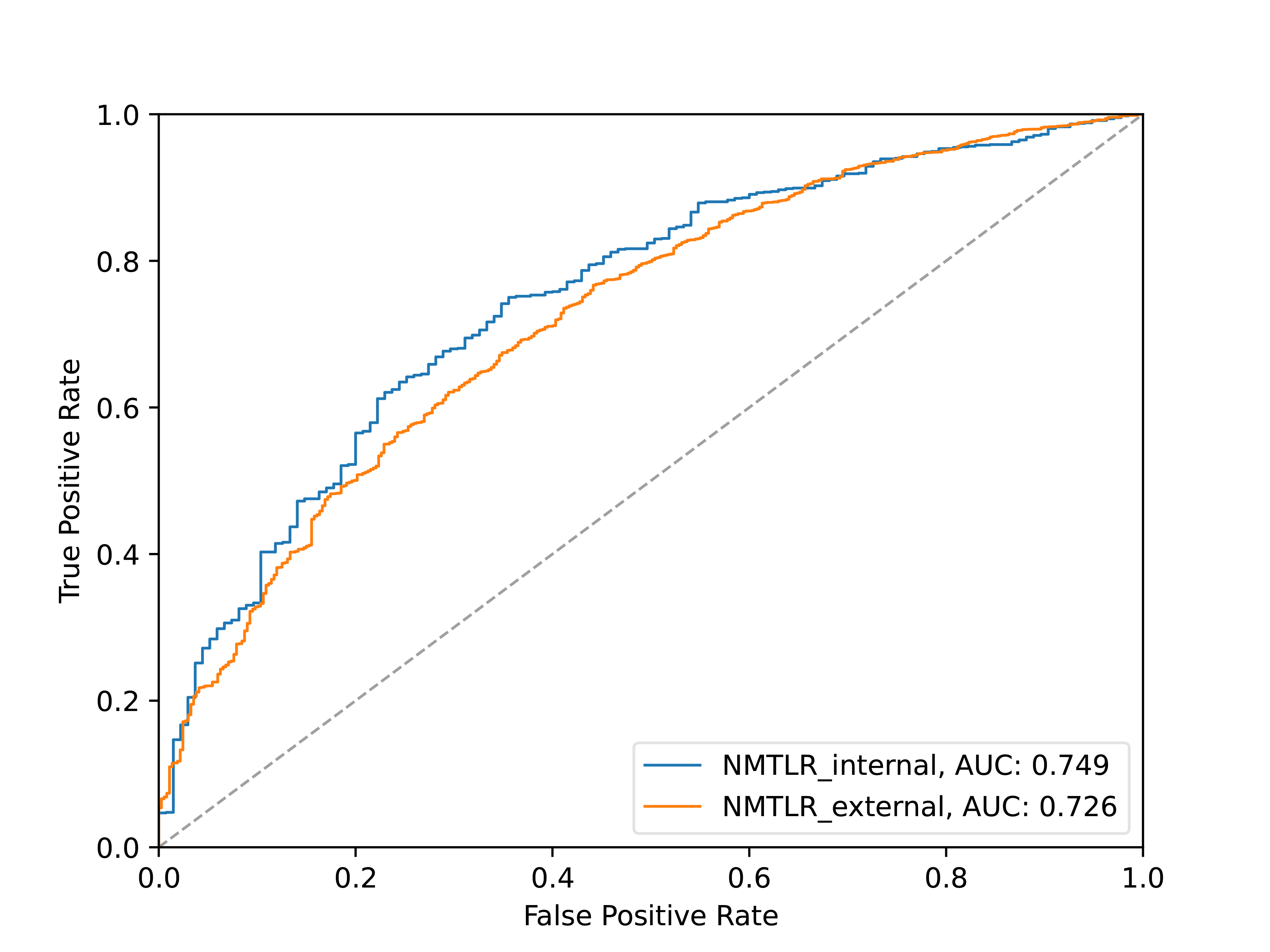


**Fig. S5**: The receiver operating curves (ROC) of 90-day readmission predictions for the compact NMTLR mode.


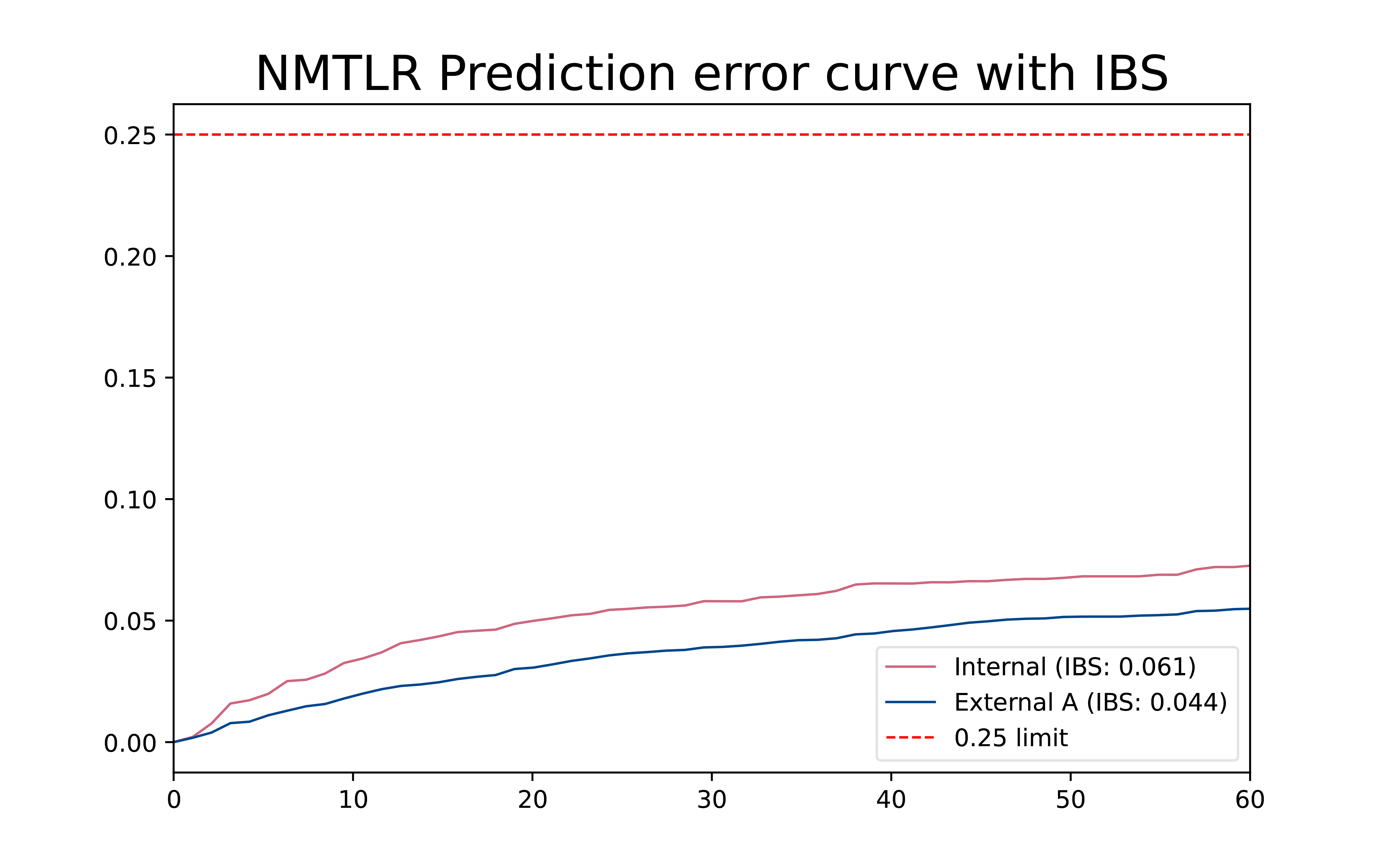


**Fig. S6**: Prediction error curves show the Brier score for the compact NMTLR mode at each time point. As a benchmark, a useful model will have a Brier score below 0.25.


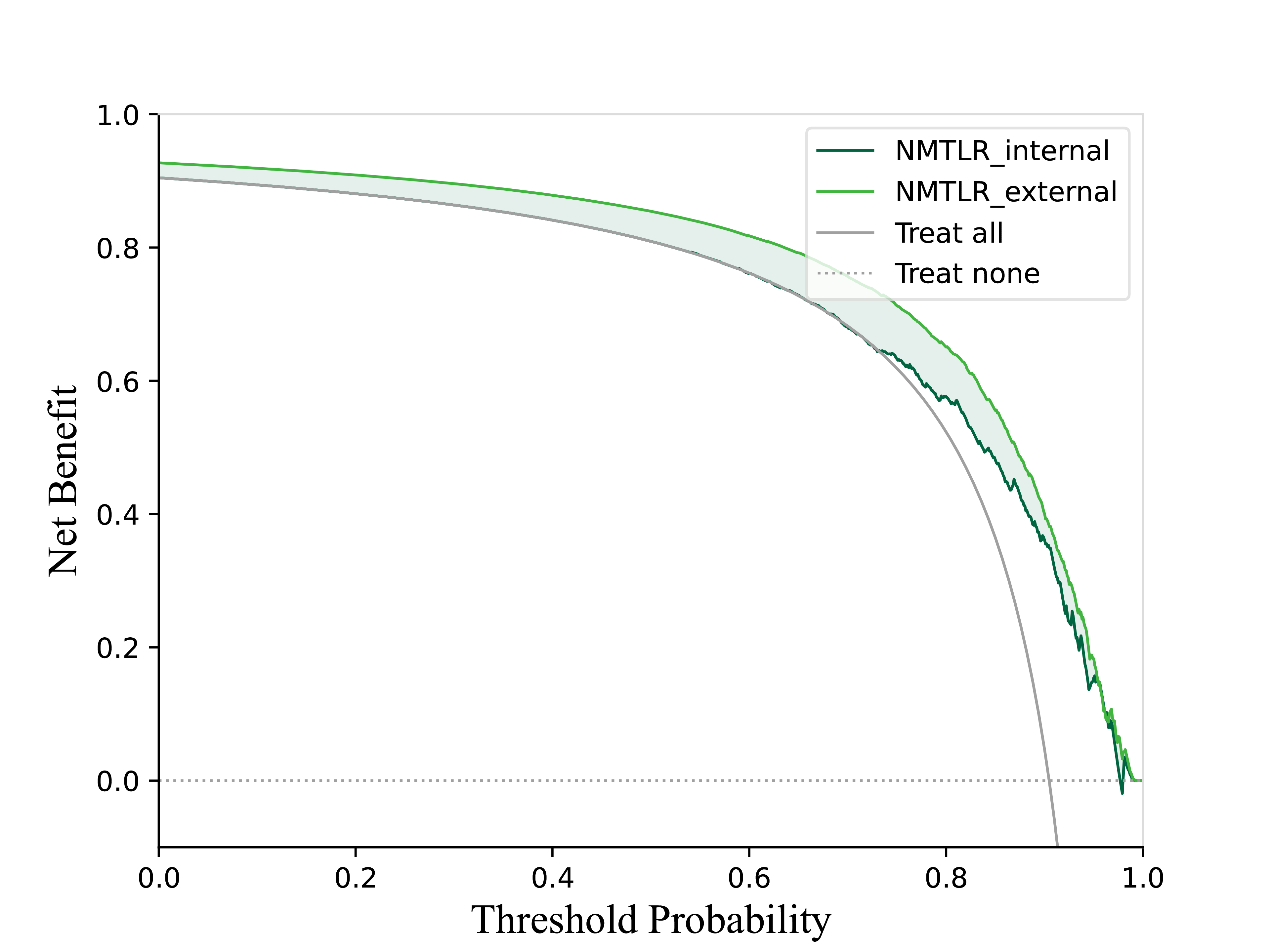


**Fig. S7**: Decision curve analysis for the compact NMTLR mode. X-axis indicates the threshold probability for critical care outcome and Y-axis indicates the net benefit. The solid gray line represents the net benefit when all patients are treated; the dashed gray line (at 0 on the y-axis) represents the net benefit when all patients are not treated.
